# Undifferentiated embryonal sarcoma of the liver in an 11-year-old boy: a case report and clinical insights

**DOI:** 10.3389/fsurg.2026.1772085

**Published:** 2026-03-06

**Authors:** Zhiru Liang, Weihong Chen, Weihong Duan, Chaoyue Song, Jianjia Xiao, Delei Yu, Yu Xie

**Affiliations:** 1 Department of Hepatobiliary Surgery, Xilingol League Central Hospital, Xilin Hot, Inner Mongolia, China; 2 Department of Central Laboratory, Anxi County Hospital, Quanzhou, Fujian, China; 3 Department of Hepatobiliary Surgery, PLA Rocket Force Characteristic Medical Center, Beijing, China; 4 Department of Medical Imaging, Xilingol League Central Hospital, Xilin Hot, Inner Mongolia, China; 5 Department of Neonatal, Anxi County Hospital, Quanzhou, Fujian, China

**Keywords:** case report, hepatectomy, liver neoplasms, pediatric oncology, undifferentiated embryonal sarcoma

## Abstract

This case report presents the clinical course and management of an 11-year-old boy who was diagnosed with undifferentiated embryonal sarcoma of the liver (UESL), a rare and aggressive pediatric malignancy. The patient was admitted with a 20-day history of right upper quadrant pain. Imaging studies revealed a large cystic-solid mass in the right hepatic lobe, suggestive of UESL, with involvement of major hepatic vessels and the diaphragm. The patient underwent a successful extended right hepatectomy (right trisectionectomy) via an anterior approach. The postoperative course was complicated by bile leakage, which resolved with conservative management. Adjuvant chemotherapy was subsequently administered. The patient recovered fully and was disease-free at follow-up. This case highlights the critical importance of a multidisciplinary approach, meticulous surgical planning, and the utility of the anterior approach for resecting large liver tumors. It also underscores the necessity of considering UESL in the differential diagnosis of pediatric liver masses and the role of multimodal therapy in achieving a favorable outcome. Clinical lessons from this case are discussed in the context of current management strategies, with emphasis on complete resection and organized post-discharge surveillance.

## 1 Introduction

Undifferentiated embryonal sarcoma of the liver (UESL) is an uncommon and highly aggressive malignant mesenchymal tumor, accounting for approximately 9% to 13% of all pediatric liver malignancies (1). It primarily affects school-aged children, with a peak incidence between 6 and 10 years of age, though cases in adolescents and adults have been rarely reported (2, 3). UESL is characterized by its rapid and relentless growth, often attaining a considerable size before clinical diagnosis is established. The clinical manifestations are often insidious and nonspecific at onset, frequently including abdominal pain or discomfort, fever, a palpable abdominal mass, and constitutional symptoms such as weight loss and anorexia (4). This nonspecific symptomatic profile, coupled with the tumor's rarity, frequently leads to delayed diagnosis or misdiagnosis, often initially mistaken for more common conditions like liver abscess or benign cystic disease (5).

Radiologically, UESL typically presents as a large, well-circumscribed, predominantly cystic-solid mass on cross-sectional imaging. The classic appearance on computed tomography (CT) and magnetic resonance imaging (MRI) is that of a complex mass with both solid enhancing components and cystic areas resulting from extensive necrosis and hemorrhage. This radiological spectrum can lead to diagnostic confusion, potentially being mistaken for benign cystic lesions (e.g., mesenchymal hamartoma) or other malignant hepatic tumors such as hepatoblastoma in younger children or hepatocellular carcinoma in specific clinical settings. Consequently, the definitive diagnosis invariably relies on histopathological examination. Microscopically, UESL is composed of densely packed, primitive spindle or stellate-shaped cells embedded within a myxoid stroma, with frequent mitotic figures and zones of necrosis (6). Immunohistochemistry plays a vital role in the differential diagnosis, typically demonstrating strong positivity for vimentin, indicating its mesenchymal origin, while being negative for hepatocytic markers (e.g., HepPar-1, Glypican-3) and alpha-fetoprotein (AFP), which helps to exclude hepatoblastoma and hepatocellular carcinoma (7).

The prognosis of UESL was historically very poor prior to the era of effective chemotherapy, owing to its aggressive behavior and high rate of local recurrence and metastasis. However, the therapeutic landscape has been transformed with the advent of multimodal therapy, which integrates complete surgical resection with intensive adjuvant chemotherapy. This combined multimodal approach has significantly improved long-term survival outcomes, with markedly better prognosis reported in contemporary case series. The optimal management requires a coordinated, multidisciplinary effort involving pediatric surgeons, oncologists, radiologists, and pathologists (8). Furthermore, for large liver tumors such as UESL, the choice of surgical approach is critical. The traditional approach to liver resection involves initial mobilization of the liver, which may lead to tumor compression, rupture, and iatrogenic dissemination. The anterior approach, which involves direct parenchymal transection without prior liver mobilization, minimizes tumor manipulation and is particularly suitable for large tumors or those in close proximity to major vascular structures (9).

We present a detailed case of UESL in an 11-year-old boy, with the aim of elucidating the comprehensive diagnostic workup, the nuances of surgical strategy including the anterior approach for a massive tumor, the management of postoperative complications, and the rationale for adjuvant chemotherapy. Through this discussion, we seek to provide valuable clinical insights and reinforce the modern principles of managing this rare but potentially curable entity.

## 2 Case report

### 2.1 Patient demographics and history

An 11-year-old boy (height 155 cm, weight 62.5 Kg) with no significant past medical history, no known drug allergies, and no family history of malignancy. He had normal growth and development. There was no history of trauma or recent travel to endemic areas. The patient presented with a 20-day history of persistent right upper quadrant pain. The pain was not associated with trauma. He had previously sought medical attention at a local hospital, where an abdominal CT scan suggested a possible hepatoblastoma. Subsequently, an abdominal MRI at another medical center revealed a large mass in the right hepatic lobe, highly suggestive of malignancy, with UESL being a primary consideration. The imaging also indicated potential involvement of the right portal branch, right and middle hepatic veins, and possible diaphragmatic invasion. During his initial hospitalization, the patient developed a high fever (peak 39.3℃) accompanied by chills, which was attributed to intratumoral hemorrhage and secondary infection. He was treated with intravenous sulbactam/cefoperazone with clinical improvement. He was then transferred to our department for further management on January 20, 2025.

#### 2.1.1 Initial examination and diagnostic workup

On admission, the patient appeared slightly pale but was conscious and well-oriented. Vital signs were stable. Abdominal examination revealed tenderness in the right upper quadrant without definite palpable mass or guarding.

#### 2.1.2 Laboratory investigations

Laboratory tests revealed anemia (hemoglobin 78 g/L) and hypoalbuminemia (albumin 29.3 g/L), with normal coagulation. Tumor markers, including alpha-fetoprotein, were within normal ranges. An elevated C-reactive protein level (61.71 mg/L) indicated an inflammatory state.

#### 2.1.3 Imaging studies

**Figure 1:**
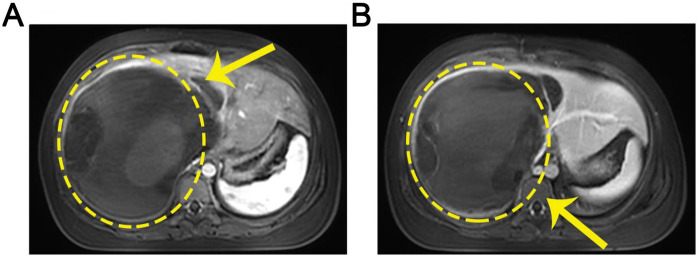
Preoperative MRI scans of the liver tumor. **(A)** Arterial phase. **(B)** Venous phase. The tumor is demarcated by yellow dotted circles in both T1-weighted images.

Preoperative B-ultrasound showed a large, well-defined cystic-solid mass measuring approximately 177 × 171 mm in the right hepatic lobe, with multiple internal septations and minimal peripheral blood flow. Preoperative MRI findings were highly characteristic of UESL (Figure 1). Liver volumetry calculated the future liver remnant (left lateral section) to be 65.8% of the standard liver volume, indicating sufficient functional reserve. The timeline of key clinical events is summarized in Table 1.

**Table 1:** Timeline of Key clinical events.

Date	Event	Key details
Early Jan 2025	Symptom onset	Right upper quadrant pain began.
Before Jan 20	Preoperative evaluation	CT and MRI indicated UESL.
Jan 20, 2025	Admission	Transferred to our center.
Jan 24, 2025	Surgery	Right trisectionectomy via anterior approach.
Jan 28, 2025	Complication	Bile leakage diagnosed.
Jan 31, 2025	Resolution	Bile leakage resolved conservatively.
Feb 1, 2025	Discharge	Patient discharged.
Mar 12, 2025	Adjuvant therapy	First cycle of IEV chemotherapy.
Jul 14, 2025	Follow-up	CT scan showed no recurrence.

#### 2.1.4 Working diagnosis and differential

Malignant liver tumor, highly suggestive of undifferentiated embryonal sarcoma. Differential diagnoses included:

Hepatoblastoma: This is the most common pediatric liver malignancy in younger children (typically <5 years). It was considered but deemed less likely due to the patient's age (11 years) and normal AFP level. Hepatoblastoma usually presents with elevated AFP in >90% of cases.

Cystic hepatoblastoma: A rare variant that can appear cystic on imaging. However, the absence of AFP elevation and the age of the patient made this less likely.

Hepatocellular carcinoma: More common in older children with underlying liver disease (e.g., hepatitis, metabolic disorders). Our patient had no such history, and imaging findings were not typical.

Hepatic hydatid disease: Although the patient had no confirmed epidemiological history, the cystic appearance on imaging warranted consideration. However, the solid components and enhancement pattern were not characteristic.

Mesenchymal hamartoma: A benign cystic liver tumor usually seen in infants. The rapid growth and symptomatic presentation in an 11-year-old were not typical.

A summary of the differential diagnosis is provided in Table 2.

**Table 2:** Differential diagnosis of pediatric liver mass in this case.

Diagnosis	Key supporting features	Key excluding features
Hepatoblastoma	Most common pediatric liver malignancy.	Patient age atypical (11 yrs); normal AFP.
Cystic Hepatoblastoma	Rare variant, cystic on imaging.	AFP normal, age atypical
Hepatocellular Carcinoma	Occurs in older children, can be primary.	No underlying liver disease; imaging atypical.
Hepatic Hydatid Disease	Cystic liver lesion.	No travel history; imaging shows solid components.
Mesenchymal Hamartoma	Benign cystic tumor in infants.	Patient age atypical; rapid, symptomatic growth.

### 2.2 Therapeutic intervention and outcomes

#### 2.2.1 Multidisciplinary team (MDT) discussion and surgical decision

MDT involving hepatobiliary surgery, oncology, radiology, pathology, anesthesiology, and transfusion medicine was convened. Key conclusions were:

The radiological features strongly suggested UESL. Preoperative biopsy was deemed high-risk due to the potential for tumor rupture and needle-track seeding.
The patient was symptomatic, nutritionally compromised, and at high risk for tumor rupture, necessitating urgent intervention.
No evidence of distant metastasis was found, making complete resection the goal.
Given the normal liver function and adequate future liver remnant, upfront surgical resection was feasible and recommended.

#### 2.2.2 Surgical planning and procedure

On January 24, 2025, the patient underwent an exploratory laparotomy and right trisectionectomy following a comprehensive preoperative imaging evaluation. Surgical planning prioritized an anterior approach to minimize tumor manipulation, with meticulous preservation of the left hepatic pedicle and left hepatic vein. The parenchymal transection was performed using an ultrasonic dissector (Harmonic ACE®) and bipolar electrocautery. Major vascular and biliary structures were ligated with silk sutures or clipped with polymer clips (Hem-o-lok®). Anticipating significant blood loss, 2,000 mL red blood cells, 2,000 mL plasma, and 200 mL platelets were prepared; however, the procedure ultimately required only 200 mL estimated blood loss without transfusion.

Intraoperatively, the liver was markedly enlarged, harboring a 17 × 18 cm giant tumor occupying the right lobe and left medial section, with firm adherence to the diaphragm. A clear ischemic line demarcated the tumor from the left lateral section, and no peritoneal or extrahepatic metastases were observed. The hepatoduodenal ligament was isolated, and a tourniquet placed for potential Pringle maneuver. The right anterior and posterior portal pedicles were individually looped and transected using a linear stapler after parenchymal division along the right falciform ligament. Critical vascular structures—including the sagittal portal vein portion, left hepatic vein, and short hepatic veins draining into the inferior vena cava—were preserved. The right triangular and hepatorenal ligaments were divided, and the tumor was removed *en bloc* with the resected liver specimen (16 × 12 × 10 cm). The diaphragm remained intact post-dissection, and the operation concluded after 3 h 20 min.

Pathological examination confirmed undifferentiated embryonal sarcoma with extensive hemorrhage and necrosis. Microscopy revealed densely packed tumor cells with marked atypia. Immunohistochemistry supported the diagnosis: Vimentin(+), CD10(+), Desmin(+), CK(focal+), CK18(+), while ARG-1(−), HepPar-1(−), GPC-3(−), AFP(−), and INI1 retained nuclear expression. The Ki-67 proliferation index was high (50%–75%).

**Figure 2:**
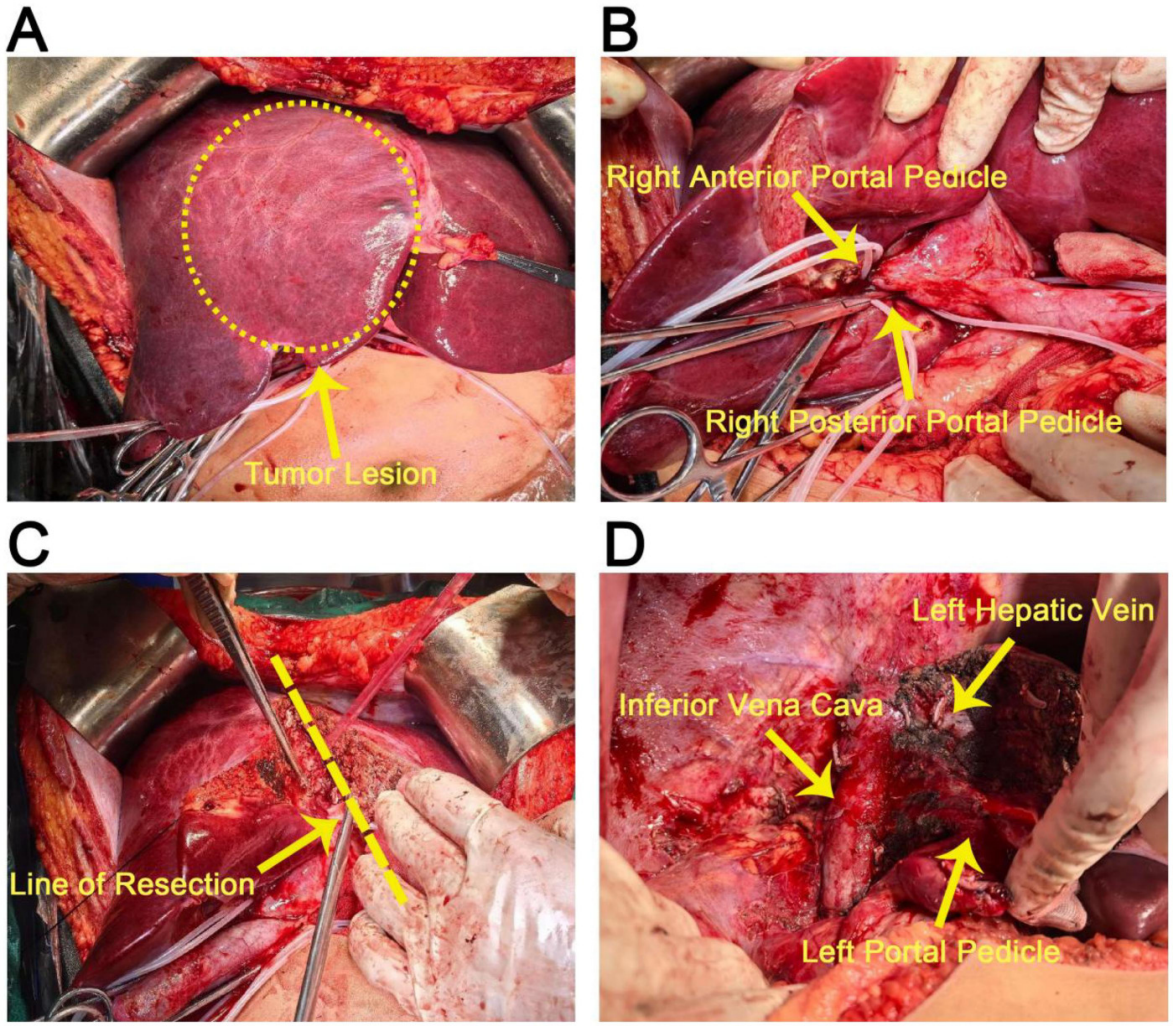
Intraoperative identification of liver tumor lesion and display of anatomical structures. **(A)** Overall view of the liver with the tumor lesion marked. **(B)** Detailed view showing the right anterior and posterior portal pedicles. **(C)** Liver resection along the determined line. **(D)** Exposure of the left hepatic vein, inferior vena cava, and left portal pedicle.

During the surgical procedure, a tumor lesion on the liver was identified (Figure 2A, marked by a yellow dotted circle). Subsequent detailed dissection revealed critical anatomical structures, including the right anterior portal pedicle and right posterior portal pedicle (Figure 2B, with labels indicating each structure). After determining the resection line, liver resection was performed (Figure 2C, showing the resection in progress). The surgical field also exposed other vital structures, namely the left hepatic vein, inferior vena cava, and left portal pedicle (Figure 2D, with each structure clearly labeled).

**Figure 3:**
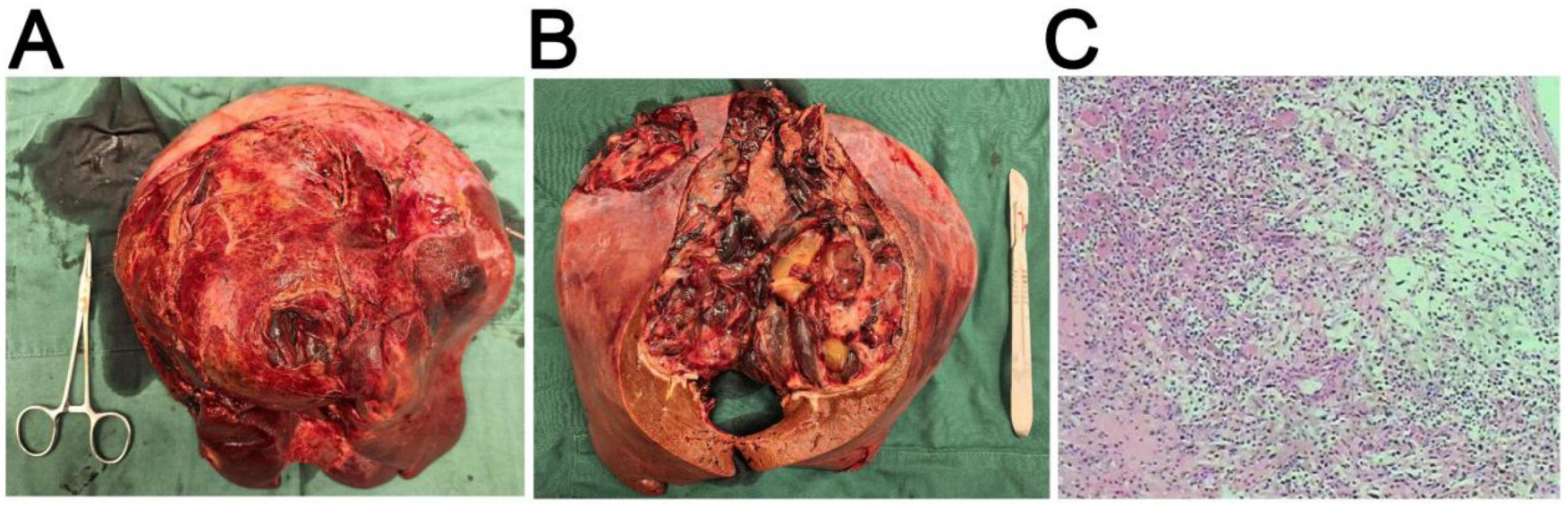
Surgically resected liver specimen and its pathological examination. **(A)** Overall view of the resected liver specimen with the tumor. **(B)** Sectioned view revealing the internal tumor structure. **(C)** Pathological examination at high magnification (×200) showing tumor cell details.

The resected liver specimen exhibited a large tumor (Figure 3A). Pathological sectioning of the specimen revealed the internal structure of the tumor (Figure 3B). Pathological examination of the resected specimen, which included immunohistochemical analysis, demonstrated densely packed tumor cells with marked atypia (Figure 3C).

**Figure 4:**
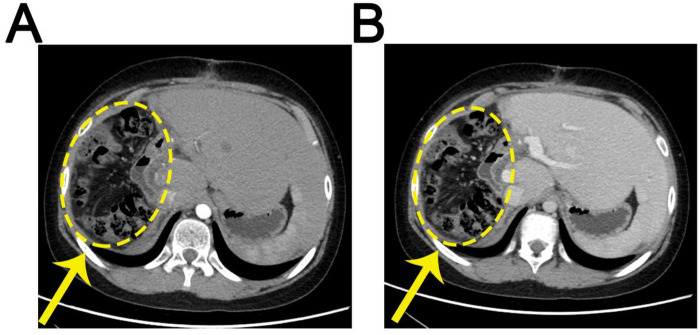
Post-operative CT scans evaluating the surgical outcome and hepatic status. **(A)** Arterial phase. **(B)** Venous phase. Both images show the resection site and surrounding hepatic structures, with the previous tumor location roughly indicated. The yellow dotted circles roughly indicate the previous tumor location.

To evaluate the surgical outcome and detect any potential distant metastases, post-operative CT scans were performed. These images, acquired in both arterial (Figure 4A) and venous phases (Figure 4B), demonstrate the post-operative state of the liver, clearly showing the resection site and surrounding structures. Providing a visual reference for assessing the extent of resection. CT's wide anatomical coverage and sensitivity for detecting metastatic lesions make it an ideal modality for post-operative surveillance.

#### 2.2.3 Postoperative management and complications

The postoperative regimen included:

Steroids: Methylprednisolone 80 mg daily for 3 days to mitigate liver ischemia-reperfusion injury and inflammation.
Fluid and Nutrition Management: A restricted fluid protocol (<1,500 mL/day) with emphasis on plasma, albumin, and glucose-insulin-potassium (GIK) solution, while limiting fat emulsions, amino acids, and saline.
The patient passed flatus on postoperative day 2 and gradually resumed oral intake.

A bile leak was detected on postoperative day 4 (January 28). This was managed conservatively with somatostatin (6 mg/day). The leak resolved completely by postoperative day 7. The patient was discharged on postoperative day 8 after suture removal. Trends in laboratory parameters (total bilirubin, ALT, PT%, albumin, WBC, hemoglobin) normalized progressively before discharge. The patient's family expressed gratitude for the multidisciplinary care and were satisfied with the treatment outcome.

#### 2.2.4 Adjuvant therapy and follow-up

Considering the tumor's large size and high Ki-67 index, adjuvant chemotherapy with the IEV regimen(Ifosfamide, Etoposide, Vindesine) was initiated 3 months post-surgery (starting March 12, 2025). The IEV regimen was selected in accordance with our institutional protocol for high-risk soft tissue sarcomas and based on literature supporting its use in UESL (10). The regimen consisted of: Etoposide (100 mg, D1-D4) + Vindesine (4.5 mg, D1, D8) + Ifosfamide (2.3 g, D1–D4). The patient tolerated the chemotherapy well and had completed 6 cycles by July 2025. A follow-up CT scan on July 14, 2025, confirmed the absence of local recurrence or distant metastasis. The patient is enrolled in a structured surveillance program (Table 3).

**Table 3:** Recommended surveillance plan.

Phase	Timeframe	Key assessments
Intensive	Years 1–2	Abdominal imaging (US/CT/MRI) & clinical exam every 3–6 months.
Regular	Years 3–5	Abdominal imaging & clinical exam every 6–12 months.
Long-term	After Year 5	Annual clinical exam; imaging as indicated.

## 3 Discussion

UESL is a rare and aggressive malignant liver tumor of childhood. Despite its rarity, it represents a significant diagnostic and therapeutic challenge due to its rapid growth and potential for misdiagnosis. The modern management of UESL relies on a multimodal approach, with complete surgical resection being the cornerstone of curative intent, followed by adjuvant chemotherapy (11).

### 3.1 Diagnostic nuances and the role of MDT

The nonspecific presentation of UESL often leads to diagnostic delay. In our patient, the initial presentation of abdominal pain and fever, coupled with a complex cystic-solid liver mass, created a broad differential diagnosis. The normal AFP level effectively ruled out hepatoblastoma, a common childhood liver tumor. The characteristic MRI findings—a large mass with cystic components, fluid-fluid levels, and peripheral/septal enhancement—are highly suggestive of UESL. The decision to forgo preoperative biopsy, driven by the risk of rupture and seeding, and to proceed directly to resection based on clinical and radiological grounds, was pivotal and supported by MDT consensus. This approach underscores the importance of a collaborative, multidisciplinary model in managing rare tumors (12).

### 3.2 Surgical strategy: the anterior approach and technical considerations

Complete surgical resection is the most critical prognostic factor for UESL. The enormous size of the tumor in this case presented a significant technical hurdle. The adoption of the anterior approach was a key strategic decision. This technique, involving initial parenchymal transection before mobilizing the right liver, minimizes rotation of the liver and compression of the inferior vena cava, thereby reducing the risk of massive hemorrhage and tumor dissemination (13). Meticulous dissection to preserve the vasculature of the left lateral section (the future liver remnant) was paramount. The successful resection with minimal blood loss (200 mL) without transfusion highlights the efficacy of precise preoperative planning and advanced surgical technique (14). This technique is particularly advocated for large or centrally located tumors to avoid rotation-related hemodynamic instability and to achieve a clear resection margin under direct vision. Our experience corroborates the findings of recent series which highlight that the anterior approach, when feasible, is associated with reduced intraoperative blood loss and enhanced safety in pediatric major hepatectomy (15).

### 3.3 Comprehensive postoperative management

The postoperative course was complicated by bile leakage, a known complication after major hepatectomy. The successful conservative management with somatostatin demonstrates that low-output biliary fistulas can often be treated non-operatively. Our postoperative fluid and nutritional strategy was designed to support liver regeneration while preventing fluid overload and edema in the vulnerable remnant. The use of short-course, high-dose methylprednisolone may have helped modulate the systemic inflammatory response post-surgery (16).

### 3.4 The evolving role of adjuvant therapy

Historically, the prognosis for UESL was dismal. The integration of combination chemotherapy, often including anthracyclines, ifosfamide, and etoposide, has dramatically improved survival rates, now exceeding 70% in many series. In this case, the IEV regimen was chosen based on its activity in soft tissue sarcomas. The decision for adjuvant therapy was based on the high-risk features of the tumor (large size, high proliferative index) (17). The IEV regimen, incorporating ifosfamide and etoposide, aligns with the backbone of many effective sarcoma protocols. Its selection reflects a tailored approach considering the tumor's high-grade morphology and size, consistent with the principle of risk-adapted therapy emphasized in contemporary guidelines. The favorable tolerance observed in our patient mirrors the manageable toxicity profile reported in pediatric cohorts receiving similar regimens (18). The absence of recurrence at 6-month follow-up is encouraging and aligns with the current paradigm of multimodal therapy.

### 3.5 Study limitations

This study has several limitations. First, as a single case report, the findings cannot be generalized. Second, the follow-up period is relatively short (6 months), and long-term outcomes are needed to confirm the efficacy of treatment. Third, we did not perform molecular profiling of the tumor, which could provide insights into tumor biology and potential targeted therapies. Fourth, some baseline patient information (e.g., detailed genetic and psychosocial history) was not available due to the acute referral nature of the case. Future studies with larger samples and longer follow-up are warranted. Furthermore, while the absence of recurrence was confirmed by radiology report, a dedicated follow-up imaging figure was not available for this report; future long-term studies will aim to include such direct visual documentation.

## 4 Conclusion

This case provides a comprehensive illustration of the successful management of a large UESL in a child. It reinforces that despite its aggressiveness, UESL is a curable disease with a multimodal strategy. Key takeaways include: (1) the central role of MDT in diagnostic and therapeutic planning; (2) the importance of aggressive, yet meticulous, surgical resection utilizing techniques like the anterior approach; (3) the need for vigilant postoperative management to handle complications like bile leak; (4) the essential contribution of adjuvant chemotherapy to long-term survival. Long-term follow-up remains mandatory to monitor for late recurrence and sequelae of treatment.

## Data Availability

The original contributions presented in the study are included in the article/Supplementary Material, further inquiries can be directed to the corresponding author/s.
